# *miR-218-5p*靶向TPX2调节p53通路影响肺腺癌恶性进展

**DOI:** 10.3779/j.issn.1009-3419.2023.101.30

**Published:** 2023-10-20

**Authors:** Jiangong XU

**Affiliations:** 453003 新乡，新乡医学院第三附属医院胸外科; Department of Thoracic Surgery, The Third Affiliated Hospital of Xinxiang Medical College, Xinxiang 453003, China

**Keywords:** 肺肿瘤, TPX2, miR-218-5p, p53, 转移, 凋亡, Lung neoplasms, TPX2, miR-218-5p, p53, Metastasis, Apoptosis

## Abstract

**背景与目的:**

肺腺癌（lung adenocarcinoma, LUAD）是肺癌的一种主要亚型，其治疗与诊断依然是目前的研究热点。靶向Xklp2靶蛋白（targeting protein for Xenopus kinesin-like protein 2, TPX2）在多种癌细胞中高表达，可能与LUAD的发生发展相关。本研究旨在探究TPX2对LUAD细胞恶性进程的影响以及调控机制。

**方法:**

通过生物信息学分析技术，对癌症基因组图谱（The Cancer Genome Atlas, TCGA）数据库中LUAD组织中基因TPX2的表达情况进行分析。实时荧光定量聚合酶链式反应（quantitative real-time polymerase chain reaction, qRT-PCR）检测人肺正常细胞系和人LUAD细胞系中TPX2和miR-218-5p的表达水平。蛋白质印迹法（Western blot）检测细胞系中TPX2蛋白表达以及其对p53信号通路关键蛋白表达的影响。使用生物信息学预测并通过双荧光素酶报告基因检测验证TPX2与miR-218-5p的关系，细胞活力检测（cell counting kit-8, CCK-8）、细胞克隆形成、细胞划痕、Transwell实验、流式细胞术检测miR-218-5p和TPX2对LUAD细胞功能的影响。

**结果:**

LUAD细胞中TPX2显著高表达，敲低TPX2可以抑制LUAD细胞的增殖、迁移、侵袭，促进凋亡并产生G_2_/M期阻滞，并且促进p53信号通路关键蛋白的表达。miR-218-5p是TPX2的上游调控因子，可以抑制其表达。过表达miR-218-5p能消除TPX2高表达导致的恶性发展，抑制LUAD细胞的增殖、迁移等恶性进程以及促进p53信号通路。

**结论:**

miR-218-5p靶向抑制TPX2表达，并通过p53发挥抑制LUAD细胞恶性发展的作用。

## Abstract (en)

**Background and objective:**

Lung adenocarcinoma (LUAD) is a major subtype of lung cancer, and its treatment and diagnosis remain a hot research topic. Targeting protein for Xenopus kinesin-like protein 2 (TPX2) is highly expressed in a variety of cancer cells and may be associated with the progression of LUAD. This study aimed to investigate the effect of TPX2 on the malignant progression of LUAD cells and the regulatory mechanisms.

**Methods:**

The expression of gene TPX2 in LUAD tissues from The Cancer Genome Atlas (TCGA) database was analyzed by bioinformatics analysis techniques. Quantitative real-time polymerase chain reaction (qRT-PCR) was used to detect the expression levels of TPX2 and miR-218-5p in human lung normal cell lines and human LUAD cell lines. Western blot was used to detect TPX2 protein expression in cell lines and its effect on the expression of key proteins in the p53 signaling pathway. The relationship between TPX2 and miR-218-5p was predicted using bioinformatics and verified by dual luciferase reporter gene assay. Cell counting kit-8 (CCK-8) assay, cell clone formation, cell scratching, Transwell assay, and flow cytometry were used to detect the effects of miR-218-5p and TPX2 on LUAD cell function.

**Results:**

TPX2 was significantly overexpressed in LUAD cells, and knockdown of TPX2 inhibited LUAD cell proliferation, migration, and invasion, promoted apoptosis and induced G_2_/M phase block, and promoted the expression of key proteins in the p53 signaling pathway. miR-218-5p, an upstream regulator of TPX2, could inhibit its expression. Overexpression of miR-218-5p eliminated the malignant development caused by high expression of TPX2, inhibited the malignant processes of LUAD cells such as proliferation and migration as well as promoted the p53 signaling pathway.

**Conclusion:**

miR-218-5p targets and inhibits TPX2 expression and exerts an inhibitory effect on the malignant progression of LUAD cells via p53.

据世界卫生组织对于2020年全球癌症发病情况的统计^[1]^，肺癌是癌症死亡的主要原因。在全球的肺癌病例中，肺腺癌（lung adenocarcinoma, LUAD）作为肺癌的一种亚型，确诊病例约占所有肺癌病例的40%^[2]^。虽然近年新的药物和治疗方法使得LUAD患者的生存情况有所改善，但由于患者被诊断时往往已为晚期以及药物抗性等原因，使得LUAD死亡率正逐年增加^[3]^。因此，深入理解LUAD调控恶性表型的机制对于药物的开发、诊断标志物的发现等十分重要和紧急。

靶向Xklp2靶蛋白（targeting protein for Xenopus kinesin-like protein 2, TPX2）是减数分裂和有丝分裂的重要调控因子。近几年来TPX2因在乳腺癌、肝细胞癌、子宫内膜癌等多种人类恶性肿瘤中出现显著高表达，被认为是潜在的癌基因^[4⇓-6]^。Li等^[7]^的研究表明TPX2有成为前列腺癌诊断和预后生物标志物的潜力。此外，TPX2与癌症的进展密切相关，例如：在胆管癌、肝细胞癌和前列腺癌中TPX2表达水平与癌细胞的上皮细胞-间充质转化（epithelial-mesenchymal transition, EMT）相关^[8⇓-10]^；在结直肠癌中TPX2高表达且促进癌症的进展^[11]^。有研究^[12]^报道TPX2可能是LUAD预后的独立因素，并可能通过参与LUAD中的几种癌症相关信号通路促进癌症发生发展。另外，有研究^[13]^表明沉默TPX2能促进LUAD细胞A549凋亡以及促进p53蛋白表达，表明TPX2可能是LUAD的促癌基因，TPX2在LUAD中的调控机制值得进一步挖掘并验证，进而明确其调控作用以及上下游调控机制。因此本研究选择TPX2作为研究对象。

本研究旨在以TPX2为出发点，深入探究其在LUAD中的上游和下游调控机制，以及其串联轴对LUAD恶性发展过程的具体作用，帮助深入理解TPX2发挥调控作用的具体机制，为寻找LUAD的靶向诊断和治疗的新方法提供一定的理论依据。

## 1 资料与方法

### 1.1 临床样本

收集2023年1月至2023年3月在新乡医学院第三附属医院胸外科手术切除的新鲜LUAD组织标本和配对癌旁组织标本各20例。患者均签署知情同意书。

### 1.2 细胞

研究所用的人肺上皮细胞系BEAS-2B细胞（CTCC-400-0007）和人LUAD细胞A549细胞（CTCC-001-0036）、A-427细胞（CTCC-001-0055）、Calu-3细胞（CTCC-003-0104）、PC-9（CTCC-400-0185）、H1975（CTCC-001-0354）均购自于美森细胞。

### 1.3 主要试剂

胎牛血清购自以色列Bioind公司；青链霉素混合液购自中国Solarbio公司；BEGM基础培养基购自瑞士Lonza公司；F-12K基础培养基、EMEM完全培养基、RPMI-1640基础培养基购自中国中乔新舟生物科技公司；Trizol、RIPA缓冲液、Lipofectamine 2000购自美国Thermo Fisher Scientific公司；miR-NC、miR-218-5p mimic、siR-TPX2、siR-NC均购自中国锐博生物；pcDNA3.1-TPX2和pcDNA3.1空白质粒均购自中国生工生物工程公司；p53抑制剂pifithrin-α（PFT-α）购自德国Merck公司；HiScript II Q RT SuperMix for qPCR、miRNA 1^st^ Strand cDNA Synthesis试剂盒购自中国诺唯赞公司；SYBR Premix Ex Taq试剂盒购自日本TaKaRa公司；细胞活力检测（cell counting kit-8, CCK-8）试剂购自中国碧云天生物技术有限公司；基质胶购自美国Corning公司；BCA蛋白检测试剂盒、超敏化学发光试剂购自中国碧云天生物技术有限公司；PAGE凝胶快速制备试剂盒购自中国雅酶公司；双荧光素酶表达载体pmirGLO-Vector、双荧光素酶检测试剂盒购自美国Promega公司；一抗TPX2、p53、p-p53（Ser15）、MDM2、Bax、GAPDH及二抗山羊抗兔IgG均购于英国Abcam公司。

### 1.4 生信分析

从肿瘤基因组图谱（The Cancer Genome Atlas, TCGA）（https://portal.gdc.cancer.gov/）中下载LUAD的miRNA成熟体（Normal: n=46, Tumor: n=521）、mRNA的表达量数据（Normal: n=59, Tumor: n=535），利用“edgeR”包对miRNA（|logFC|>1, Padj<0.05）、mRNA（|logFC|>2, Padj<0.05）进行Normal组和Tumor组的差异分析获得差异miRNA及差异mRNA，通过文献参考确定目标基因mRNA。利用starBase（http://starbase.sysu.edu.cn/）、mirDIP（http://ophid.utoronto.ca/mirDIP/index.jsp#r）数据库预测目标基因mRNA的上游调控基因，获得与目标基因mRNA靶向结合位点的差异miRNA。通过GSEA软件对目标基因mRNA进行通路富集分析，研究目标mRNA的下游效应物。

### 1.5 免疫组化

将LUAD和癌旁临床组织样本固定包埋并切片后，4 ^o^C下与一抗TPX2（1:1000）孵育过夜，随后加入二抗孵育30 min，采用苏木素染核，梯度酒精脱水，树脂封片。倒置显微镜观察并拍照。

### 1.6 细胞培养

人支气管上皮细胞BEAS-2B在添加了10%胎牛血清和1%青链霉素混合液的BEGM中培养；LUAD细胞A549在添加了10%胎牛血清和1%青链霉素混合液的F12K中培养；LUAD细胞A-427、H1975、PC-9在添加了10%胎牛血清和1%青链霉素混合液的RPMI-1640中培养。所有细胞置于37 ^o^C、5% CO_2_的培养箱中进行培养。

### 1.7 细胞转染和处理

使用miR-218-5p mimic、siR-TPX2、pcDNA3.1-TPX2和各自对照序列或质粒以及A549和PC-9细胞系，按照Lipofectamine 2000转染试剂说明书构建各转染组：siR-TPX2、siR-NC、miR-218-5p mimic、miR-NC、miR-218-5p+oe-TPX2、miR-NC+oe-TPX2、miR-NC+oe-NC。另外使用DMSO溶解p53抑制剂（PFT-α）按照说明书配比进行溶解，将转染好的细胞培养24 h后添加PFT-α，在20 μmol/L的浓度下处理1 h。构建正常表达TPX2的细胞（siR-NC+DMSO）、敲低TPX2的细胞（siR-TPX2+DMSO）及敲低TPX2的同时抑制p53发挥作用的细胞（siR-TPX2+PFT-α）。

### 1.8 蛋白免疫印迹（Western blot）

采用添加了蛋白酶抑制剂的RIPA裂解缓冲液提取细胞样品的总蛋白。采用BCA试剂盒测定样品蛋白浓度。将等量的蛋白使用10%的SDS-PAGE分离，随后将蛋白电转至PVDF膜上。使用5%脱脂牛奶封闭2 h，4 ^o^C条件下与兔抗TPX2（1:1000）、兔抗MDM2（1:1000）、兔抗p-p53（磷酸化S15，1:5000）、兔抗p53（1:1000）、兔抗Bax（1:2000）、兔抗GAPDH（1:10,000）进行孵育过夜。经TBST清洗再与山羊抗兔IgG（1:2000）在室温下孵育2 h。使用超敏化学发光液处理后在化学发光成像系统下观察。GAPDH为内参蛋白。

### 1.9 总RNA提取和实时荧光定量聚合酶链式反应（quantitative real-time polymerase chain reaction, qRT-PCR）

使用Trizol提取组织和细胞的总RNA，在Nanodrop 2000分光光度计中检测RNA浓度。使用HiScript II Q RT SuperMix for qPCR试剂盒合成对mRNA进行逆转录，使用miRNA 1^st ^Strand cDNA Synthesis试剂盒对miRNA进行逆转录。SYBR Premix Ex Taq用于qPCR检测，miR-218-5p以U6为内参，TPX2以GAPDH为内参。qPCR使用的引物序列见表1。

**表1:** qRT-PCR引物序列

Gene	Sequences	
miR-218-5p	Forward primer	5'-CGAGTGCATTTGTGCTTGATCTA-3'
	Reverse primer	5'-TAATGGTCGAACGCCTAACGTC-3'
U6	Forward primer	5'-CTCGCTTCGGCAGCACA-3'
	Reverse primer	5'-AACGCTTCACGAATTTGCGT-3'
TPX2	Forward primer	5'-AACCACCCACCGAGCCTAT -3'
	Reverse primer	5'-CACCCACAACATCTTCCAAAA-3'
GAPDH	Forward primer	5'-TGCACCACCAACTGCTTA-3'
	Reverse primer	5'-GATGCAGGGATGATGTTC-3'

qRT-PCR: quantitative real-time polymerase chain reaction.

### 1.10 细胞增殖实验

将单细胞悬液接种在96孔板中（3000个/孔），补加培养液至100 μL，在37 ^o^C、5% CO_2_的培养箱中培养。分别于0、24、48、72、96 h后加入10 μL CCK-8溶液，避光孵育2 h后使用酶标仪检测450 nm处的吸光度。

### 1.11 克隆形成实验

各处理组细胞分别接种于6孔板中（1000个/孔）。在37 ^o^C、5% CO_2_的培养箱中培养14 d。弃培养基并洗涤后，用4%多聚甲醛固定细胞15 min，弃固定液，加适量0.05%结晶紫染15 min，清洗后干燥；观察克隆情况并拍照、计数。

### 1.12 划痕愈合实验

各处理组细胞分别接种于6孔板中（5×10^4^个/孔），在37 ^o^C、5% CO_2_培养箱中培养至约占90%。以直尺作辅助，使用200 μL的枪头在每个孔的中间划一条线。用磷酸盐缓冲液（phosphate buffered saline, PBS）洗去划下的细胞，加入无血清培养基。拍照记录培养0和24 h后的划痕宽度。

### 1.13 Transwell细胞侵袭实验

将用无血清培养基重悬的细胞接种于铺有Matrigel基质胶的Transwell小室中（2×10^4^个/孔）。在下室添加含10% FBS的培养基。于37 ^o^C、5% CO_2_的培养箱培养24 h后，用棉棒除去未侵袭的细胞和基底膜基质，使用结晶紫对膜下方的细胞进行染色，显微镜下拍照并记录个数。

### 1.14 细胞凋亡及周期流式分析

（1）细胞凋亡：细胞用4^ o^C的PBS洗涤后，在避光条件下，使用PI和FITC-Annexin V对细胞进行染色15 min。最后使用流式细胞仪分析细胞凋亡。（2）细胞周期：细胞中加入预冷的70%乙醇进行固定，于4 ^o^C放置3-4 h后去除乙醇，加入PI和RNA酶，37 ^o^C、避光孵育15 min，然后使用流式细胞仪进行细胞周期分析。

### 1.15 双荧光素酶报告实验

在两个pmirGLO双荧光载体中分别插入TPX2的3’UTR片段和突变的TPX2 3’UTR片段，将重组质粒转染至A549细胞中，分别为TPX2-WT和TPX2-MUT。TPX2-WT和TPX2-MUT各分为两组，将miR-218-5p mimic和miR-NC分别转染至TPX2-WT或TPX2-MUT的两组细胞中。培养48 h后使用双荧光素酶检测试剂盒检测荧光素酶活性，以海肾荧光素酶活性作参照。

### 1.16 统计学分析

使用SPSS 16.0进行统计分析，数据采用均数±标准差（Mean±SD）表达，两组间比较使用t检验，P<0.05为差异有统计学差异。采用GraphPad Prism 8.0.2使检测数据可视化。

## 2 结果

### 2.1 TPX2在LUAD中表达量分析

从TCGA中下载LUAD的mRNA表达量数据，通过差异分析后获得2503个在Tumor组和Normal组之间存在表达差异的mRNA，见图1A。生信分析TCGA数据库中LUAD数据集显示TPX2在LUAD组织中显著高表达，见图1B。qRT-PCR分析得LUAD组织中TPX2表达量显著高于正常组织（P<0.001），见图1C。免疫组化结果显示，20例LUAD组织中TPX2蛋白水平均高于对应的癌旁正常组织，见图1D。qRT-PCR检测得人LUAD细胞A549、H1975、PC-9、H1299中TPX2相对表达量显著高于BEAS-2B细胞（P<0.05），见图1E。Western blot结果见图1F，可见LUAD细胞中TPX2蛋白水平高于BEAS-2B细胞。选择相对表达较显著的A549和PC-9细胞进行后续实验。

**图1:**
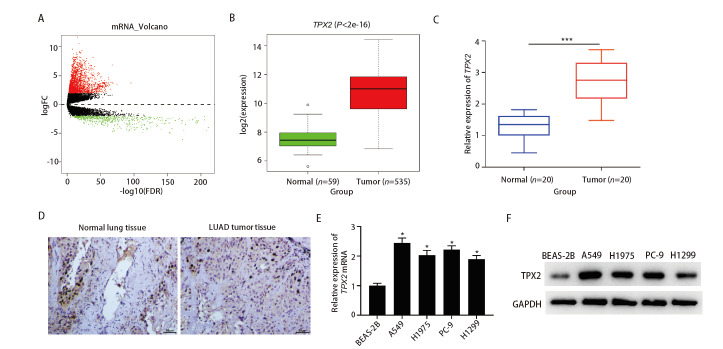
TPX2在LUAD中高表达。A：LUAD mRNA表达差异分析的火山图；B：LUAD数据集中TPX2的差异表达情况；C：qRT-PCR检测LUAD组织和癌旁组织临床样本中TPX2 mRNA的表达水平；D：免疫组化检测LUAD组织和癌旁组织临床样本中TPX2蛋白表达的代表性结果（×200）；E：qRT-PCR检测BEAS-2B、A549、H1975、PC-9、H1299细胞中TPX2 mRNA的表达水平；F：Western blot检测TPX2在5种细胞系中的蛋白水平。*：与BEAS-2B组相比，P<0.05；***：与Normal组相比，P<0.001。

### 2.2 siR-NC组与siR-TPX2组细胞增殖、迁移、侵袭能力和凋亡率、细胞周期分布情况比较

qRT-PCR结果显示siR-TPX2-1组、siR-TPX2-2组的A549和PC-9细胞mRNA相对表达量均显著低于siR-NC组（P<0.05），Western blot实验结果显示siR-TPX2-1组和siR-TPX2-2组的A549和PC-9细胞TPX2蛋白表达量显著低于siR-NC组，见图2A。选择表达量差异最显著的siR-TPX2-2进行后续的功能研究。CCK-8结果显示siR-TPX2组A549和PC-9细胞活性显著低于siR-NC组（P<0.05），见图2B。克隆形成、划痕愈合、Transwell、细胞凋亡实验结果见图2C-图2F，与siR-NC组相比，siR-TPX2组A549和PC-9细胞增殖、迁移、侵袭能力显著降低，凋亡率显著升高（P<0.05）。细胞周期分析结果显示，相比于siR-NC组，siR-TPX2组A549和PC-9细胞G_0_/G_1_期细胞显著减少，细胞均显著阻滞于G_2_/M期（P<0.05），见图2G。

**图2:**
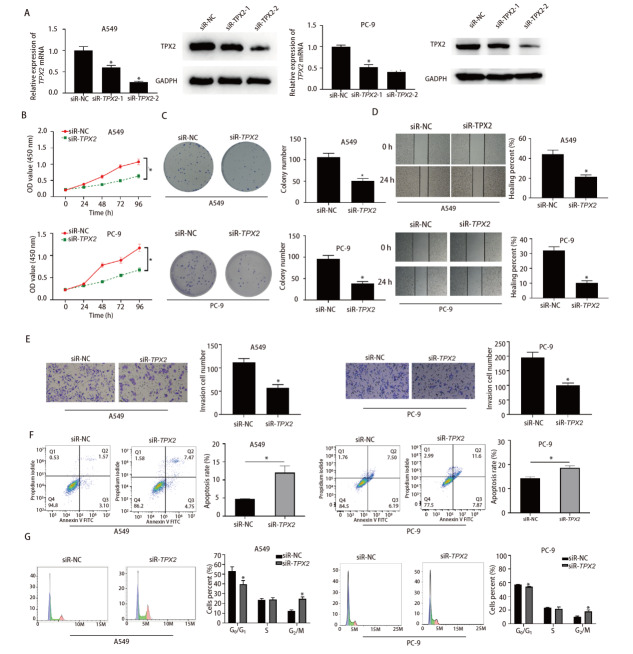
下调TPX2会抑制LUAD细胞增殖、转移并促进细胞凋亡的发生。A：qRT-PCR和Western blot检测A549细胞中TPX2的mRNA和蛋白表达水平的下调情况；B, C：CCK-8增殖试验和克隆形成试验检测A549细胞增殖能力的变化；D：划痕愈合试验检测A549细胞迁移能力的变化；E：Transwell试验检测A549细胞侵袭能力的变化；F：流式细胞术检测A549细胞凋亡率的变化；G：流式细胞术检测A549细胞周期分布的变化。*：与siR-NC组相比，P<0.05。

### 2.3 TPX2和p53信号通路关系分析

为进一步挖掘TPX2发挥调控功能的下游效应器，我们通过GSEA富集得到TPX2显著参与p53等信号通路，见图3A。Western blot检测A549细胞p53信号通路相关蛋白p53、MDM2、磷酸化p53（p-p53, Ser15）和Bax，结果显示沉默TPX2能有效地增加p53蛋白表达水平、p53 Ser15磷酸化水平、Bax蛋白以及MDM2的表达水平，但是这种作用效果会被PFT-α消除，而PFT-α不会影响TPX2的表达水平，见图3B。CCK-8结果显示，siR-TPX2+DMSO组的A549和PC-9细胞活力均显著低于siR-NC+DMSO组（P<0.05），而siR-TPX2+PFT-α组则显著高于siR-TPX2+DMSO组（P<0.05），见图3C。划痕愈合、Transwell、细胞凋亡实验结果见图2D-图2F，与siR-NC+DMSO组相比，siR-TPX2+DMSO组A549和PC-9细胞克隆、迁移、侵袭能力显著降低，凋亡率显著升高（P<0.05），而与siR-TPX2+DMSO组相比，siR-TPX2+PFT-α组细胞克隆、迁移、侵袭能力显著提高，凋亡率显著降低（P<0.05）。细胞周期分析显示相比于siR-NC+DMSO组，siR-TPX2+DMSO组A549和PC-9细胞均显著阻滞于G_2_/M期（P<0.05），而siR-TPX2+PFT-α组的G_0_/G_1_期细胞显著增加（P<0.05）。

**图3:**
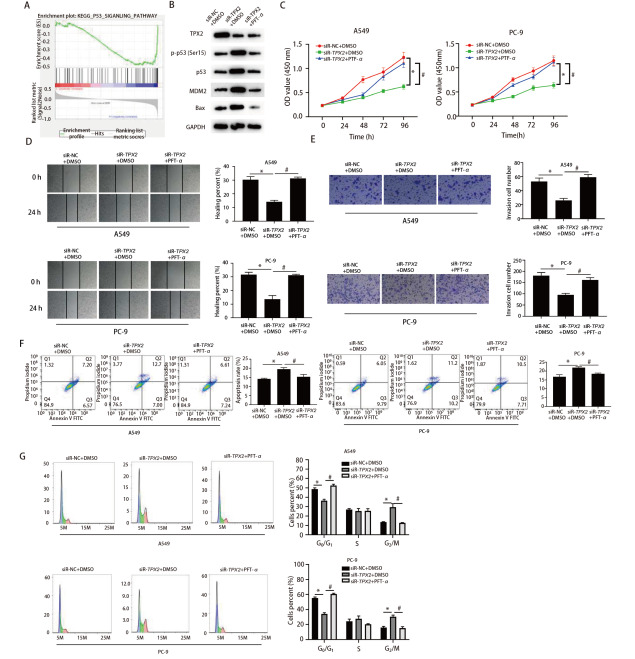
TPX2能够调控p53信号通路。A：对TPX2进行GSEA富集的结果；B：Western blot检测A549细胞中TPX2蛋白和p53信号通路相关蛋白Bax、MDM2、p53和p-p53的表达水平；C：CCK-8检测A549和PC-9细胞增殖能力的变化；D：划痕愈合试验检测A549和PC-9细胞迁移能力的变化；E：Transwell试验检测A549和PC-9细胞侵袭能力的变化；F：流式细胞术检测A549和PC-9细胞侵袭能力的变化；G：流式细胞术检测A549和PC-9细胞的细胞周期分布。*：与siR-NC+DMSO组相比，P<0.05；^#^：与siR-TPX2+DMSO组相比，P<0.05。

### 2.4 在LUAD中TPX2的表达量与miR-218-5p表达量相关性

为发现TPX2的上游调控因子，本研究从TCGA中得到LUAD的miRNA表达量数据，通过差异分析后获得299个存在表达差异的miRNA，见图4A。将该组差异表达中下调的miRNA和利用starBase、mirDIP数据库对TPX2进行上游调控基因预测得到的miRNA进行对照，取三者的交集发现4个与TPX2有靶向结合位点的差异miRNA，见图4B。对TPX2和4个差异miRNA作Pearson相关性分析，发现miR-218-5p是其中与TPX2负相关程度最高的，见图4C、图4D。对LUAD的数据集进行差异分析发现，miR-218-5p在LUAD组织中显著低表达，见图4E。通过对5个细胞系进行qRT-PCR的检测，我们进一步发现在LUAD细胞中miR-218-5p表达水平下调（P<0.05），见图4F。

**图4:**
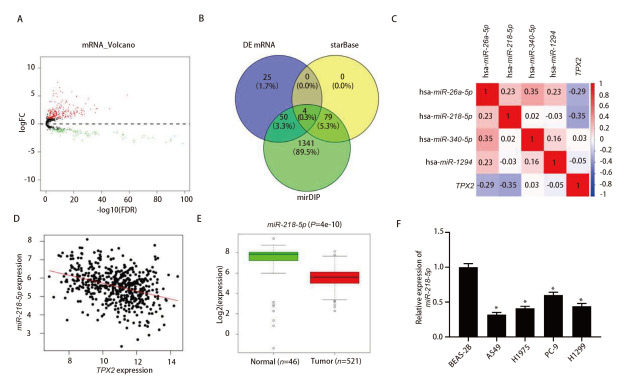
miR-218-5p在LUAD中低表达且与TPX2呈负相关。A：LUAD中miRNA表达差异分析的火山图；B：starBase、mirDIP数据库预测的TPX2上游调控基因和下调miRNA集之间的交集；C：TPX2与交集中miRNAs的Pearson相关性分析；D：miR-218-5p和TPX2的Pearson相关性分析；E：miR-218-5p在LUAD数据集中的差异表达情况；F：qRT-PCR检测miR-218-5p在BEAS-2B、A549、H1975、PC-9、H1299细胞中的表达水平。*：与BEAS-2B组相比，P<0.05。

### 2.5 TPX2与miR-218-5p的靶向关系

qRT-PCR检测显示miR-218-5p mimic组细胞miR-218-5p的表达水平显著高于miR-NC组（P<0.05），见图5A。通过starBase数据库预测得到TPX2和miR-218-5p具有靶向结合位点，见图5B。双荧光素酶报告实验结果显示miR-218-5p mimic组细胞中野生型TPX2报告基因的荧光活性显著低于miR-NC组（P<0.05）；突变型TPX2报告基因的荧光活性与miR-NC组比较，差异无统计学意义（P>0.05），见图5C。qRT-PCR检测得miR-218-5p mimic组细胞TPX2表达水平显著低于miR-NC组（P<0.05）。Western blot检测得miR-218-5p mimic组细胞TPX2蛋白表达水平显著低于miR-NC组（P<0.05），见图5D。

**图5:**
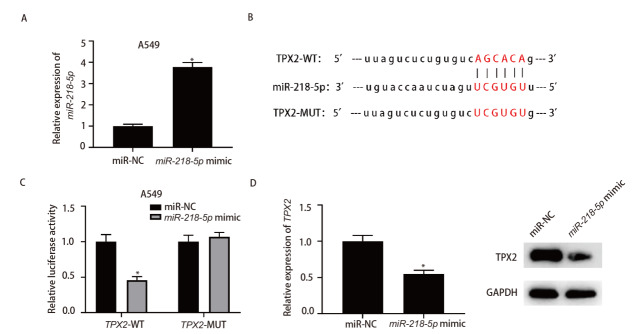
miR-218-5p通过靶向结合TPX2的3’UTR调控TPX2的表达水平。A：qRT-PCR检测转染了miR-NC和miR-218-5p mimic的A549细胞中miR-218-5p的表达水平；B：预测miR-218-5p与TPX2的结合位点，并改变位点序列以获得TPX2-MUT；C：双荧光素酶报告实验检测miR-218-5p与TPX2的结合关系；D：qRT-PCR和Western blot检测TPX2表达的变化。*：与miR-NC组相比，P<0.05。

### 2.6 miR-NC+oe-NC组、miR-NC+oe-TPX2组、miR-218-5p+oe-TPX2组细胞增殖、迁移、侵袭能力和凋亡率、细胞周期分布情况比较

qRT-PCR和Western blot检测miR-NC+oe-NC组、miR-NC+oe-TPX2组、miR-218-5p+oe-TPX2组A549和PC-9细胞TPX2表达量，miR-NC+oe-TPX2组的TPX2表达量显著高于siR-NC组，而miR-218-5p+oe-TPX2组TPX2表达量显著低于miR-NC+oe-TPX2组（P<0.05），见图6A。CCK-8结果显示miR-NC+oe-TPX2组A549和PC-9细胞活力显著高于miR-NC+oe-NC组，miR-218-5p+oe-TPX2组细胞活力显著低于miR-NC+oe-TPX2组（P<0.05），见图6B。划痕愈合、Transwell、细胞凋亡实验结果见图6C-图6E。与miR-NC+oe-NC组相比，miR-NC+oe-TPX2组A549和PC-9细胞迁移、侵袭能力显著增强，凋亡率显著降低，而与miR-NC+oe-TPX2组相比，miR-218-5p+oe-TPX2组细胞迁移、侵袭能力显著降低，凋亡率显著增高（P<0.05）。细胞周期分析结果显示相比于miR-NC+oe-NC组，miR-NC+oe-TPX2组A549和PC-9细胞G_0_/G_1_期细胞，而相比于miR-NC+oe-TPX2组，miR-218-5p+oe-TPX2组G_0_/G_1_期细胞显著减少，细胞显著阻滞于G_2_/M期（P<0.05），见图6F。Western blot检测p53信号通路相关的蛋白p53、磷酸化p53（Ser15）、MDM2和Bax的表达，见图6G，发现上调miR-218-5p能够消除过表达TPX2导致的Bax、MDM2、p53的表达水平和磷酸化p53（Ser15）水平的下调。

**图6:**
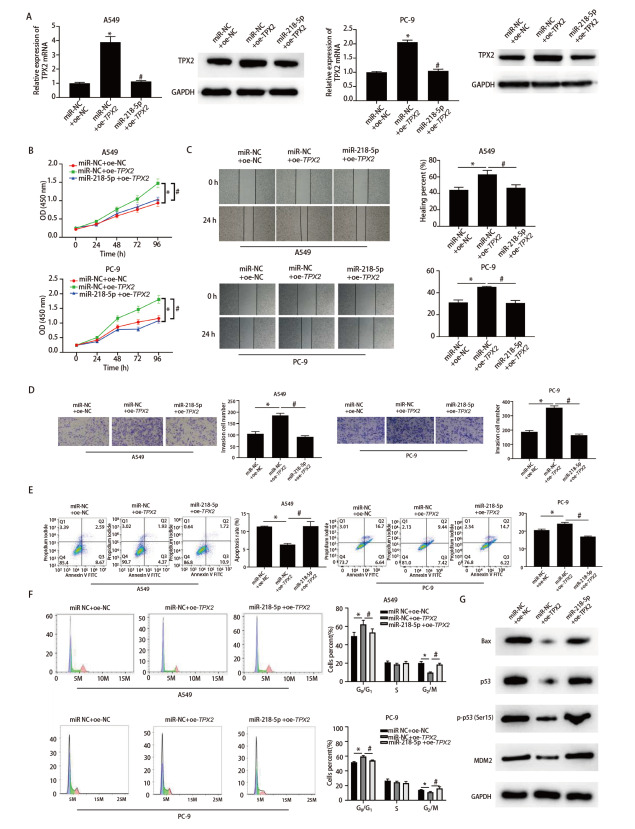
miR-218-5p靶向TPX2从而通过p53信号通路调控LUAD细胞的恶性表型。A：qRT-PCR和Western blot检测TPX2在A549和PC-9细胞中的表达水平；B：CCK-8检测A549和PC-9细胞增殖能力的变化；C：划痕愈合试验检测A549和PC-9细胞迁移能力的变化；D：Tanswell侵袭实验检测细胞侵袭能力的变化；E：流式细胞术检测A549和PC-9细胞的凋亡率；F：流式细胞术检测A549和PC-9细胞凋亡率的变化；G：Western blot检测A549细胞中p53、磷酸化p53（Ser15）、MDM2和Bax的表达变化。*：与miR-NC+oe-NC组相比，P<0.05；#：与miR-NC+oe-TPX2组相比，P<0.05。

## 3 讨论

全球死亡率最高的肺癌中LUAD有很大占比^[1,2]^。本研究通过生物信息学对LUAD差异基因的分析发现，TPX2基因在LUAD组织中高表达。已有的研究^[14]^表明TPX2在多种癌症（膀胱尿路上皮癌、肝细胞癌、LUAD、胃腺癌）中高表达，可作为癌症的预后分子生物标志物和治疗这些疾病的潜在治疗靶点。本研究中的实验结果也表明TPX2在LUAD中高表达，对LUAD的恶性进展起促进作用。对TPX2进行通路富集分析后发现其显著参与p53信号通路。大量的研究表明p53行使抑癌作用。p53信号通路主要包含p53和MDM2。MDM2为p53的E3泛素连接酶，通过将p53泛素化使其降解，而p53作为一个激活MDM2表达的转录因子，在降解后形成一个负反馈，二者因此达成平衡。而在细胞存在压力时，二者平衡被打破，p53表达量上调，引发凋亡、细胞周期分布改变等活动^[15]^。刘等^[13]^研究表明沉默TPX2能促进LUADA549细胞的凋亡，其作用可能与上调p53表达和下调Bcl-2表达有关。本研究结果同样显示TPX2具有促癌的作用，与p53信号通路的功能相左，因此后续通过PFT-α抑制p53信号通路，并敲低TPX2进行回复实验，验证了TPX2可通过抑制p53信号通路，从而促进LUAD细胞增殖、迁移和侵袭并抑制凋亡。

本研究中我们的实验结果表明TPX2可以抑制p53信号通路，并且敲低TPX2能够增加磷酸化p53（Ser15）的蛋白水平，结合过去关于结肠癌的研究^[16]^显示p53的Ser15磷酸化能够增加p53的稳定性，防止其p53被MDM2降解，蛋白印记法检测p53信号通路相关蛋白的结果显示TPX2和p53信号通路之间具体的调控机制可能是通过改变p53磷酸化情况从而改变p53的稳定性，进而影响p53信号通路及下游的基因表达。此外，TPX2、MDM2和p53三者被发现存在直接的相互作用^[17]^，与本研究中敲低TPX2能够提高p53和MDM2表达量的结果相照应，TPX2、MDM2和p53复合体的形成也可能是TPX2与p53信号通路的互作方式。以上研究结果提示TPX2可能通过多条路径与p53信号通路产生互作。

随着对miRNA的研究逐渐增加，众多科研团队发现其在癌症发展中发挥重要作用^[18]^。本研究通过生物信息学发现TPX2的上游调控miRAN miR-218-5p，并通过双荧光素报告实验证明二者存在结合关系。在以往的研究^[19]^中，miR-218-5p被发现在多种癌症中具有调控作用，例如，在宫颈癌的研究^[20]^中，miR-218-5p通过LYN/核因子-κB（nuclear factor kappa-B, NF-κB）抑制癌细胞的生长和转移。在乳腺癌中，miR-218-5p通过靶向结合CX43 mRNA抑制乳腺癌细胞的增殖、迁移，并减弱细胞的吉西他滨抗药性。值得注意的是，Chen等^[21]^研究表明，miR-218-5p在LUAD中低表达且能够和mRNA ERO1A结合发挥抑癌的作用。这些研究说明miR-218-5p能够结合多个靶基因对癌细胞的多个恶性表型进行调控。本研究发现miR-218-5p能够通过靶向结合TPX2下调TPX2的表达水平，抑制LUAD的增殖、迁移、侵袭，并促进凋亡和G_2_/M期阻滞的发生。

综上，本研究揭示了miR-218-5p/PX2/p53信号通路的互作轴，并且发现其在LUAD中参与恶性进程的调控，串联TPX2发挥调控作用的信号通路，该调控机制可能为LUAD提供诊断和预后标志物，帮助挖掘潜在的癌症发展相关因子，并为LUAD提供可能的治疗方案，如恢复miR-218-5p的表达、抑制TPX2的表达以及修复p53的正常功能。本研究结论有待在动物实验中进行进一步验证。

**Competing interests**

The author declare no competing interests.

## References

[1] Sung H, Ferlay J, Siegel RL, et al. Global Cancer Statistics 2020: GLOBOCAN estimates of incidence and mortality worldwide for 36 cancers in 185 countries. CA Cancer J Clin, 2021, 71(3): 209-249. doi: 10.3322/caac.21660 33538338

[2] Denisenko TV, Budkevich IN, Zhivotovsky B. Cell death-based treatment of lung adenocarcinoma. Cell Death Dis, 2018, 9(2): 117. doi: 10.1038/s41419-017-0063-y 29371589 PMC5833343

[3] Zhao J, Li L, Wang Q, et al. CircRNA expression profile in early-stage lung adenocarcinoma patients. Cell Physiol Biochem, 2017, 44(6): 2138-2146. doi: 10.1159/000485953 29241190

[4] Wang T, Zhang F, Zhang P. Role of the TPX2/NCOA5 axis in regulating proliferation, migration, invasion and angiogenesis of breast cancer cells. Exp Ther Med, 2023, 25(6): 304. doi: 10.3892/etm.2023.12003 PMC10203914 37229326

[5] Wang H, Chen W, Qi Y, et al. miR-29c suppresses the malignant phenotype of hepatocellular carcinoma cells in vitro by mediating TPX2 associated with immune infiltration. Dig Dis Sci, 2023, 68(5): 1923-1935. doi: 10.1007/s10620-022-07810-3 36583803

[6] Wang J, Zheng H, He H, et al. TPX2 serves as a cancer susceptibility gene and is closely associated with the poor prognosis of endometrial cancer. Genet Res (Camb), 2022, 2022: 5401106. doi: 10.1155/2022/5401106 35356748 PMC8942693

[7] Li W, Xu W, Sun K, et al. Identification of novel biomarkers in prostate cancer diagnosis and prognosis. J Biochem Mol Toxicol, 2022, 36(9): e23137. doi: 10.1002/jbt.23137 35686336

[8] Zou Z, Zheng B, Li J, et al. TPX2 level correlates with cholangiocarcinoma cell proliferation, apoptosis, and EMT. Biomed Pharmacother, 2018, 107: 1286-1293. doi: 10.1016/j.biopha.2018.08.011 30257343

[9] Liang B, Jia C, Huang Y, et al. TPX2 level correlates with hepatocellular carcinoma cell proliferation, apoptosis, and EMT. Dig Dis Sci, 2015, 60(8): 2360-2372. doi: 10.1007/s10620-015-3730-9 26025609

[10] Zhang B, Zhang M, Li Q, et al. TPX2 mediates prostate cancer epithelial-mesenchymal transition through CDK1 regulated phosphorylation of ERK/GSK3beta/SNAIL pathway. Biochem Biophys Res Commun, 2021, 546: 1-6. doi: 10.1016/j.bbrc.2021.01.106 33556637

[11] Taherdangkoo K, Kazemi Nezhad SR, Hajjari MR, et al. miR-485-3p suppresses colorectal cancer via targeting TPX2. Bratisl Lek Listy, 2020, 121(4): 302-307. doi: 10.4149/BLL_2020_048 32356447

[12] Huo C, Zhang MY, Li R, et al. Comprehensive analysis of TPX2-related ceRNA network as prognostic biomarkers in lung adenocarcinoma. Int J Med Sci, 2020, 17(16): 2427-2439. doi: 10.7150/ijms.49053 33029085 PMC7532481

[13] Liu Y, Zhang H, Tang X. TPX2 gene-targeted short hairpin RNA induces the apoptosis of human lung adenocarcinoma A549 cells and its related mechanism. Zhongliu, 2011, 31(12): 1055-1060.

[14] Shao T, Jiang X, Bao G, et al. Comprehensive analysis of the oncogenic role of targeting protein for Xklp 2 (TPX2) in human malignancies. Dis Markers, 2022, 2022: 7571066. doi: 10.1155/2022/7571066 36304254 PMC9596273

[15] Mukherjee N, Bhunia D, Garai PK, et al. Designed novel nuclear localizing anticancer peptide targets p 53 negative regulator MDM2 protein. J Pept Sci, 2023: e3535. doi: 10.1002/psc.3535 37580909

[16] Son Y, An Y, Jung J, et al. Protopine isolated from Nandina domestica induces apoptosis and autophagy in colon cancer cells by stabilizing p53. Phytother Res, 2019, 33(6): 1689-1696. doi: 10.1002/ptr.6357 30932278

[17] Chen M, Zhang H, Zhang G, et al. Targeting TPX2 suppresses proliferation and promotes apoptosis via repression of the PI3k/AKT/P 21 signaling pathway and activation of p53 pathway in breast cancer. Biochem Biophys Res Commun, 2018, 507(1-4): 74-82. doi: 10.1016/j.bbrc.2018.10.164 30454896

[18] Shi Y, Liu Z, Lin Q, et al. MiRNAs and cancer: key link in diagnosis and therapy. Genes (Basel), 2021, 12(8): 1289. doi: 10.3390/genes12081289 34440464 PMC8395027

[19] Xu Y, He Q, Lu Y, et al. MicroRNA-218-5p inhibits cell growth and metastasis in cervical cancer via LYN/NF-kappaB signaling pathway. Cancer Cell Int, 2018, 18: 198. doi: 10.1186/s12935-018-0673-1 30524205 PMC6278036

[20] Xia C, Jiang H, Ye F, et al. The multifunction of miR-218-5p-Cx43 axis in breast cancer. Onco Targets Ther, 2019, 12: 8319-8328. doi: 10.2147/OTT.S218524 31632081 PMC6790128

[21] Chen G, Wang Q, Wang K. MicroRNA-218-5p affects lung adenocarcinoma progression through targeting endoplasmic reticulum oxidoreductase 1 alpha. Bioengineered, 2022, 13(4): 10061-10070. doi: 10.1080/21655979.2022.2063537 35441565 PMC9161986

